# Post-pandemic resurgence of *Mycoplasma pneumoniae* in coastal China: from seasonal waves to sustained transmission and expanded age susceptibility

**DOI:** 10.1186/s12879-026-12790-0

**Published:** 2026-02-07

**Authors:** Jinwei Zhu, Suqing Wu, Tianfu Xu, Bijuan Zheng, Yan Chen, Yushan Zhuang

**Affiliations:** 1 Pediatrics Department, Affiliated Hospital of Putian University, Section 2, Putian, China; 2 Department of Ultrasound, Affiliated Hospital of Putian University, Putian, China; 3 Department of Neonatology, Affiliated Hospital of Putian University, Putian, China; 4 Department of Laboratory Medicine, Affiliated Hospital of Putian University, Putian, China

**Keywords:** Epidemiology, *Mycoplasma pneumoniae*, Pediatrics, Correlation analysis

## Abstract

**Background:**

Mycoplasma pneumoniae (MP) represents a primary etiological agent of pediatric Acute Respiratory Infections (ARIs) in China. However, the epidemiological landscape of MP in coastal regions during the post-COVID-19 era remains poorly characterized. Here, we delineate the epidemiological dynamics of MP—including trends, seasonality, age-specific distribution, and viral co-detection patterns—among children with ARIs in the coastal city of Putian, Southeast China.

**Methods:**

We retrospectively analyzed a cohort of 10,193 pediatric patients hospitalized with ARIs between December 2022 and November 2024. Oropharyngeal swabs from this cohort were assayed for MP and a panel of other respiratory pathogens using a multiplex reverse transcription-polymerase chain reaction (RT-PCR) platform. Data were stratified by time, season, and age to delineate the epidemiological characteristics of MP positivity.

**Results:**

Across the surveillance period (Dec 2022–Nov 2024), the overall MP positivity rate was 33.7% (3,437/10,193), with a significant increase from 28.3% in the first year to 37.5% in the second (*P* < 0.001), signaling a major post-pandemic resurgence. The epidemic pattern shifted from a discrete seasonal wave in the first year to a sustained, high-level plateau of transmission in the second. While school-age children consistently exhibited the highest positivity rates (59.1%), the age distribution of MP cases broadened significantly to include a larger proportion of younger children over the study period. Viral co-detections were identified in 25.5% of MP-positive cases, with the prevalence of co-detection being inversely correlated with age. Although the overall co-detection rate remained stable, MP-HAdV co-detections increased significantly in the second year (*P* < 0.001). Notably, the rate of co-detection was strongly correlated with the community circulation of other respiratory viruses (*R* = 0.85, *P* < 0.001), rather than the prevalence of MP itself.

**Conclusions:**

Our data indicate that the post-pandemic epidemiology of MP in this coastal Chinese region was characterized by a shift from seasonal outbreaks to prolonged, high-level transmission, and notably, a broadening of the age distribution with expanded susceptibility among younger children. This downward shift deviates from the traditional concentration in school-aged populations and may reflect the evolving epidemiological dynamics of respiratory pathogens in the post-COVID-19 landscape. To the best of our knowledge, our findings offer regional insights that may help inform surveillance efforts and guide the development of age-targeted prevention strategies.

**Clinical trial number:**

Not applicable.

## Background

The post-pandemic era has been defined by a dramatic global resurgence of *Mycoplasma pneumoniae* (MP), a principal etiological agent of pediatric acute respiratory infections that has profoundly reshaped established epidemiological patterns and challenged global public health preparedness [1–3]. In China, this substantial pediatric burden is compounded by a profound [4], yet poorly characterized, regional heterogeneity in MP dynamics [5]. Existing surveillance data reveal starkly divergent seasonal peaks—from winter-spring in the north to summer in the west [6, 7], thus rendering national, monolithic control strategies ineffective.

However, epidemiological surveillance in key southeastern coastal cities has revealed a critical knowledge gap concerning MP transmission within this densely populated, subtropical corridor [5, 8, 9]. The unique climatic milieu of these regions, including prolonged periods of warmth and humidity [5, 9], represents a critical void in our understanding of MP transmission dynamics. Whether these environmental factors drive distinct epidemic patterns is a question of urgent public health importance, particularly as emerging evidence suggests the post-pandemic resurgence involves fundamental shifts in both seasonality and age-specific susceptibility [5, 8].

To address this critical knowledge gap, we conducted a two-year surveillance study of 10,193 pediatric ARIs patients in Putian, a sentinel city within this understudied coastal region. This study was designed to delineate the contemporary epidemiological signature of MP in this unique setting by characterizing its temporal trends, age-related risk profiles, and viral co-detection landscape in the post-pandemic era. Our findings establish a crucial epidemiological benchmark for understanding and mitigating the evolving threat of MP in subtropical coastal zones globally, informing public health strategies worldwide.

## Methods

### Study design and ethical considerations

This retrospective surveillance study was conducted at the Affiliated Hospital of Putian University. This facility serves as the exclusive tertiary pediatric medical center for a catchment population of approximately 3.2 million within the subtropical coastal region of Fujian, China. The study protocol was approved by the Institutional Review Board of the Affiliated Hospital of Putian University (Approval No.2024201). Due to the retrospective nature of the study, which utilized pre-existing clinical data without influencing patient management, the Institutional Review Board waived the requirement for individual informed consent. All patient data were anonymized and de-identified prior to analysis to protect patient privacy. The study encompassed two complete surveillance years, from 1 December 2022 to 30 November 2024.

### Data collection and inclusion criteria

Pediatric patients (aged 0–14 years) were eligible for inclusion if they were hospitalized with a clinical diagnosis of ARIs. Operationally, ARIs was defined in accordance with World Health Organization (WHO) guidelines [10], characterized by the acute onset of at least one respiratory symptom (e.g., cough, sore throat, dyspnea, or tachypnea) with or without fever. A symptom duration of ≤ 14 days prior to admission was established as an inclusion criterion.

Clinical and demographic data were extracted from the hospital’s electronic medical record (EMR) system using a standardized extraction protocol. Information regarding symptom onset and duration was specifically retrieved from the ‘Chief Complaint’ section. These clinical records were subsequently merged with laboratory pathogen detection results using unique admission numbers as the common identifier. Finally, all personal identifiers were permanently removed to generate a strictly de-identified dataset for statistical analysis.

Exclusion criteria included: (1) symptom duration > 14 days; (2) presence of underlying chronic respiratory diseases (e.g., asthma, bronchopulmonary dysplasia) that could confound the assessment of an acute infection; or (3) hospitalization for a respiratory infection within the preceding 14 days.

### Pathogen detection

Oropharyngeal swabs were collected upon admission as part of standard routine clinical care to guide diagnosis and treatment. Specimen collection was performed by physicians who had undergone standardized training and obtained certification for sampling operations. It is important to note that pathogen detection was performed prospectively in real-time during the patient’s hospitalization; this study involves the retrospective analysis of these clinical laboratory results, and no stored biological samples were thawed or re-tested for the purpose of this research.

The collection procedure strictly adhered to the “Technical Guidelines for the Collection of Novel Coronavirus Pharyngeal Swabs” issued by the National Health Commission of China. The specimen type and collection protocol remained consistent throughout the study period (December 2022–November 2024). Samples were transported to the clinical laboratory under cold-chain conditions (2–8 °C). Total nucleic acids (DNA and RNA) were extracted from samples using a GenePure 96 automated system (BIOER, Hangzhou, China) according to the manufacturer’s protocols.

A commercial multiplex reverse transcription-PCR (RT-PCR) assay combined with capillary electrophoresis (13 Respiratory Pathogen Multiplex Detection Kit, Health Gene Tech, Ningbo, China; NMPA Registration No. 20183400518) was employed for the simultaneous detection of 13 respiratory pathogens, including MP. This assay utilizes an integrated one-step reaction system containing both reverse transcriptase and specific DNA polymerase. The protocol involves an initial reverse transcription step (50 °C for 15 min) to convert viral RNA into cDNA, followed by high-temperature denaturation and PCR cycling. This allows for the co-amplification of cDNA targets (from RNA viruses) and genomic DNA targets (from M. pneumoniae and Adenovirus) within a single reaction tube. PCR amplification was performed on a LongGene A300 Thermal Cycler. Subsequent amplicon identification was conducted via fragment length analysis using capillary electrophoresis on a 3500 Dx Genetic Analyzer (Thermo Fisher). Internal controls (Human DNA and RNA) were included in each run to ensure assay validity.

To ensure assay validity, three internal controls were included in each reaction: (1) Human DNA to assess sampling adequacy; (2) Human RNA to monitor RNA extraction integrity; and (3) an RT-PCR Internal Control to evaluate amplification efficiency and rule out inhibition. As this platform utilizes capillary electrophoresis, results were determined based on fluorescence peak height and fragment size rather than Cycle Threshold (Ct) values. According to the manufacturer’s instructions, a result was defined as positive if the specific pathogen peak height exceeded the Standard Positive reference peak (PosS). Peaks falling between the Standard Negative reference peak and PosS were classified as “grey zone” (low-level detection) and subjected to re-extraction and re-testing; persistent signals in this range were interpreted as positive. The analytical limit of detection for MP was validated at 5,000 copies/mL.

The multiplex panel targeted the following pathogens: human parainfluenza viruses (HPIV), Influenza B virus (IVB), human adenoviruses (HAdV), *Mycoplasma pneumoniae* (MP), *Chlamydia pneumoniae* (CP), human rhinovirus (HRV), human bocavirus (HBoV), human metapneumovirus (HMPV), human coronaviruses (HCoV), Influenza A virus (IVA), and human respiratory syncytial virus (HRSV).

### Interpretation of pathogen detection and definitions

In this study, pathogen identification was based on the detection of specific nucleic acids via RT-PCR. To ensure clinical utility, test results were reported to the treating clinicians within 24 h of specimen collection. This rapid turnaround allowed the results to directly guide therapeutic decisions. Given that the study population comprised exclusively hospitalized pediatric patients with acute respiratory symptoms (onset ≤ 14 days), detected organisms were considered clinically relevant. A “co-detection” was operationally defined as the simultaneous identification of MP and at least one other respiratory pathogen in the same clinical specimen. Due to the qualitative nature of the commercial assay, no cycle threshold cut-off values were applied to differentiate colonization from infection. In routine clinical settings, such results are typically interpreted in conjunction with clinical symptoms.

### Statistical analysis

All statistical analyses were performed using R software (version 4.4.3). Statistical significance was defined as a two-sided *P*-value < 0.05. Continuous variables were expressed as median and interquartile range (IQR) and compared using the Mann-Whitney U test. Categorical variables were summarized as frequencies and percentages (n, %) and compared using the Pearson’s χ² test or Fisher’s exact test, as appropriate. For post-hoc pairwise comparisons of categorical variables (e.g., across age and seasonal groups), the significance threshold was adjusted using the Bonferroni correction (α’ < 0.008). Spearman’s rank correlation was used to assess monotonic relationships between monthly rates of MP positivity, overall viral positivity, and MP-viral co-detection.

A restricted cubic spline (RCS) regression with five knots at predefined percentiles was employed to model the non-linear relationship between patient age and the odds of MP positivity. The number of knots was selected by comparing model fit statistics, specifically the Akaike Information Criterion (AIC) and Bayesian Information Criterion (BIC), for knots ranging from 3 to 10. Five knots were chosen as this specification minimized the BIC, providing the optimal balance between goodness-of-fit and model parsimony. Finally, a multivariable logistic regression model was constructed to quantify the adjusted association between potential risk factors (year, season, age group, gender) and MP positivity. Results were presented as adjusted odds ratios (aORs) with corresponding 95% confidence intervals (CIs).

## Results

### Post-pandemic resurgence and escalating MP burden

**Fig. 1:**
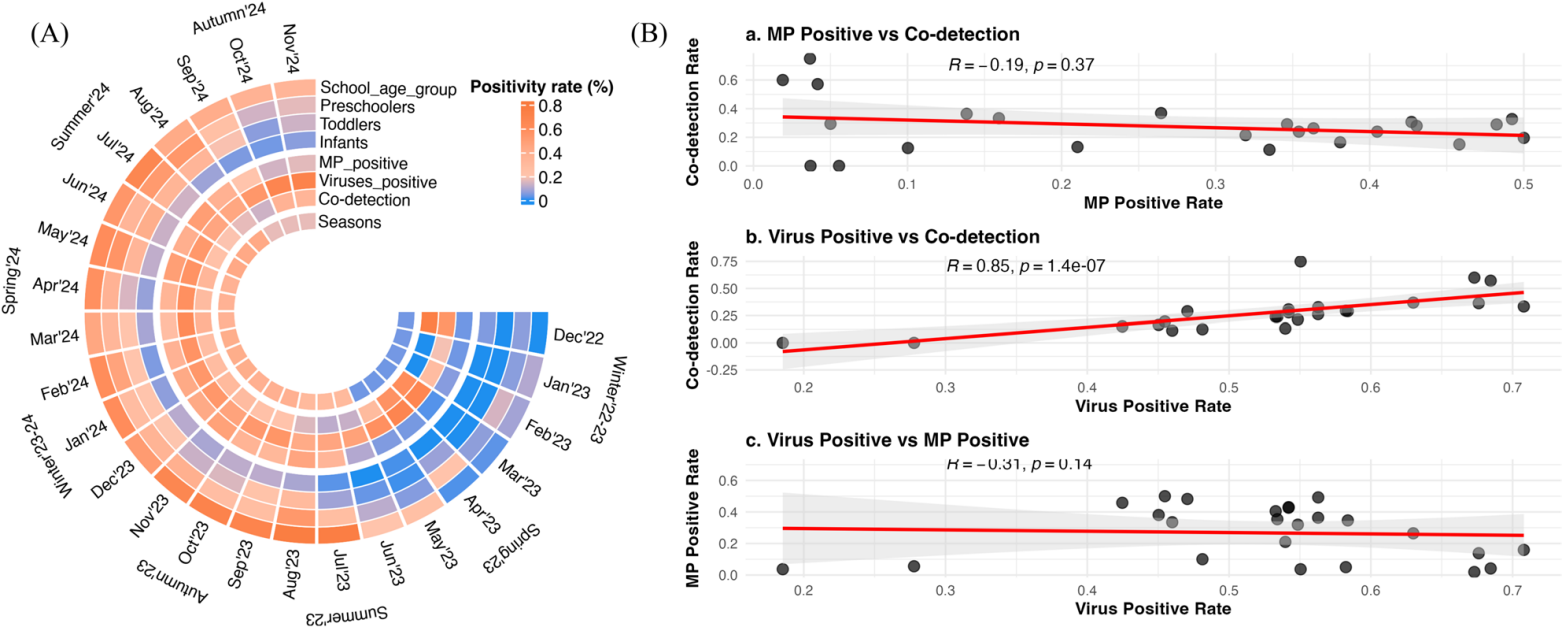
Seasonal dynamics and correlations of MP and viral detections. (**A**) Circular heatmap showing monthly positivity rates for MP, viruses, and their co-detection from December 2022 to November 2024. The color intensity corresponds to the positivity rate according to the provided scale. Seasons are indicated on the outer circumference. Rings depict overall rates and MP rates stratified by age group. (**B**)Scatter plots showing correlations between: (a) MP positive rate and co-detection rate; (b) virus positive rate and co-detection rate; and (c) virus positive rate and MP positive rate. Red lines represent the linear regression fit, with the 95%(CI) shown in grey. Spearman’s rank correlation coefficient (R) and P values are indicated for each plot

During the two-year surveillance period (Dec 2022–Nov 2024), we analyzed 10,193 pediatric patients with ARIs, identifying an overall MP positivity rate of 33.7% (3,437/10,193). This surveillance documented a significant post-pandemic resurgence, with the overall positivity rate increasing from 28.3% in the 2022–2023 period to 37.5% in the 2023–2024 period (*P* < 0.001, Table 2). This escalating burden commenced in July 2023, marking the onset of a sustained period of high-level transmission in which monthly positivity rates consistently exceeded 25%, a stark contrast to the pre-surge baseline of < 15% (Fig. 1).

**Table 1:** Demographic and clinical characteristics of participants by MP test result

Characteristics	Total Cohort (*N* = 10193)	MP-Positive (*N* = 3437)	MP-Negative (*N* = 6756)	Statistic	*P*-value
Age, median (IQR), y	4.3 (1.0–7.0)	6.1 (4.0–8.0) ^i^	3.4 (1.0–5.0)	U = 5,890,648	< 0.001
Gender, n (%)				χ^2^ = 10.031	0.002
Male	5930 (58.1)	1925 (56.0)	4005 (59.3)		
Female	4263 (41.8)	1512 (44.0)	2751 (40.7)		
Age group, n (%)				χ^2^ = 1650.795	< 0.001
Infants (< 1y)	1713 (16.8)	122 (3.5)	1591 (23.5)		
Toddlers (1-2y)	1945 (19.0)	346 (10.1) ^a^	1599 (23.7)		
Preschoolers (3-5y)	2989 (29.3)	938 (27.3) ^ab^	2051 (30.4)		
School-age (≥ 6y)	3546 (34.7)	2031 (59.1) ^abc^	1515 (22.4)		
Season, n (%)				χ^2^ = 80.753	< 0.001
Winter	2165 (21.2)	812 (23.6)	1353 (20.0)		
Spring	2673 (26.2)	731 (21.3) ^a^	1942 (28.7)		
Summer	2733 (26.8)	1026 (29.8) ^ab^	1707 (25.3)		
Autumn	2622 (25.7)	868 (25.3) ^abc^	1754 (26.0)		
Clinical Severity, n (%)				χ² = 241.696	< 0.001
ICU Admission	555 (5.4)	26 (0.8)	529 (7.1)		
General Ward	9638(94.6)	3411 (99.2)	6227 (92.9)		
Intervention, n (%)				χ² = 298.541	< 0.001
Bronchoscopy	283 (2.8)	234 (6.8)	49 (0.7)		
Not Performed	9910 (97.2)	3203 (93.2)	6707 (99.3)		

**Notes**: Statistical comparisons between MP-Positive and MP-Negative groups were performed using the Mann-Whitney U test for age and the Chi-square test for categorical variables; a *P*-value < 0.05 was considered significant for these overall comparisons. Superscripts denote a statistically significant difference for pairwise comparisons within the MP-Positive column only, based on post-hoc Chi-square tests. The significance threshold for these post-hoc tests was set at a Bonferroni-corrected *P*-value < 0.008

For Age group: ᵃ *P* < 0.008 vs. Infants.ᵇ *P* < 0.008 vs. Toddlers.ᶜ *P* < 0.008 vs. Preschoolers. For Season: ᵃ *P* < 0.008 vs. Winter. ᵇ *P* < 0.008 vs. Spring. ᶜ *P* < 0.008 vs. Summer

### Transformation of epidemic dynamics from a seasonal wave to a sustained plateau

Although aggregate data suggested bimodal summer and winter peaks (Table 1), a stratified year-over-year analysis revealed a profound transformation in epidemic dynamics. The surveillance period commenced with low baseline activity in Winter 2022–2023 (4.0%). Subsequently, the epidemic exhibited a sharp escalation, surging to high positivity in Autumn 2023 (38.8%). Unlike typical seasonal outbreaks that recede rapidly, this surge merged into a prolonged epidemic plateau, with persistently high transmission across Winter (40.8%), Spring (36.9%), and Summer (43.6%) (Table 2; Fig. 1). This sustained high-level circulation eventually showed signs of recession in the final surveillance season, with positivity declining to 16.8% in Autumn 2024. This transition from a seasonal outbreak to extended high-level circulation was further corroborated by multivariable logistic regression, which demonstrated a marked attenuation of seasonality as a risk factor for MP positivity in the second surveillance period (Fig. 3).

### Broadening of the age-specific risk profile to encompass younger children

**Fig. 2:**
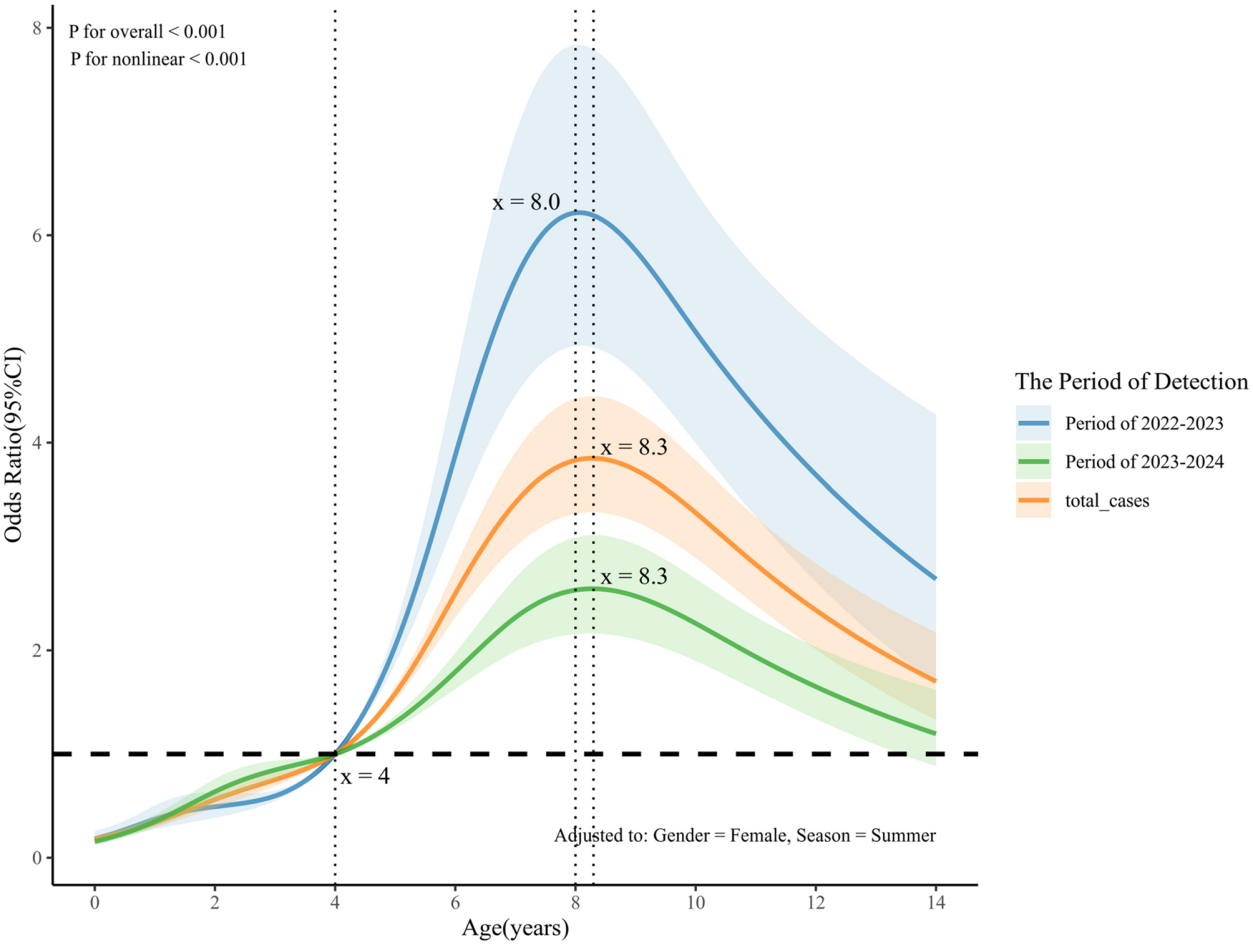
Age-dependent, non-linear risk of MP detection positivity. Five knots (k = 5) were selected based on the lowest BIC and were placed at the 10th, 25th, 50th, 75th, and 90th percentiles. The X-axis denotes age (years), while the Y-axis represents the odds ratio (OR) with corresponding 95% CIs for MP detection positivity

**Fig. 3:**
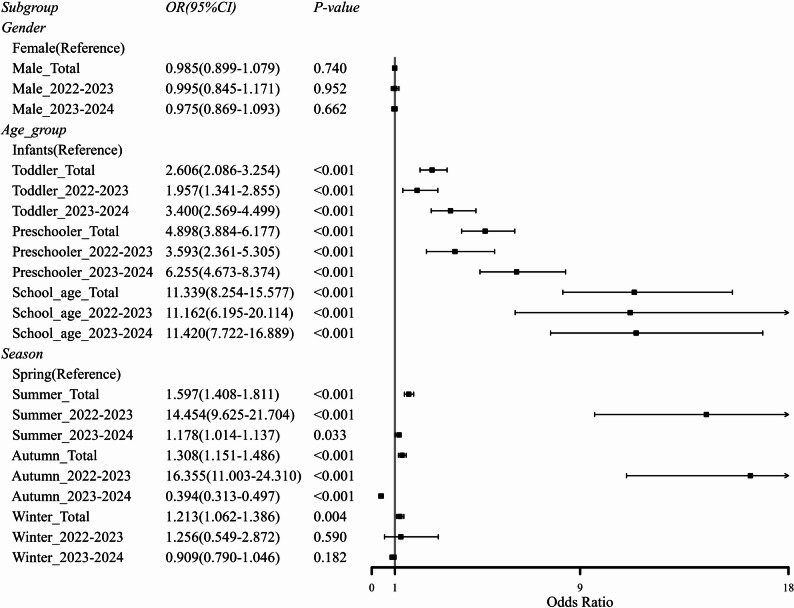
Forest plot of subgroup analysis for MP detection positivity risk. The forest plot presents subgroup analysis results for MP detection positivity risk, stratified by gender, age group, and season. The reference groups include females (gender), infants (age group), and spring (season). Statistically significant differences are determined by *P*-values (< 0.05)

A strong age-dependent susceptibility was observed, with MP positivity increasing monotonically from 7.1% in infants to a peak of 59.1% in school-age children (≥ 6 years) (Table 1). Restricted cubic spline modeling revealed that the odds of MP detection increased sharply after age 4, peaking at 8.3 years (Fig. 2). Critically, this age-specific risk profile evolved significantly between the two surveillance years. The risk curve for 2023–2024 was substantially flattened and exhibited a leftward shift compared to the 2022–2023 period, indicating a broadening of risk to younger age groups (Fig. 2). This temporal shift was quantified in our multivariable model, which revealed a dramatic increase in the adjusted odds of MP detection for toddlers and preschoolers in the second year relative to the first, while the risk for school-age children remained stable at a high level (Fig. 3).

**Table 2:** Characteristics of MP positivity in patients with ARIs during the 2022–2023 and 2023–2024 surveillance periods

Characteristics	2022–2023 *N* = 4210 (%)	2023–2024 *N* = 5983 (%)	Statistic	*P*-value
Total. Positive, n/N (%)	1191/4210 (28.3)	2246/5983 (37.5)	χ^2^ = 94.604	< 0.001
Age, median (IQR), y	6.2 (4.0–8.0)	6.0 (4.0–8.0)	U = 1,393,320	0.043
Gender, n/N (%)			χ^2^ = 105.130	< 0.001
Male	677/2463 (27.5)	1248/3467 (35.9)		
Female	514/1747 (29.4)	998/2516 (39.6)		
Age group, n/N (%)			χ^2^ = 1650.795	< 0.001
Infants	44/750 (5.9)	78/963 (8.0)		
Toddlers	112/941 (11.9)	234/1004 (23.3)		
Preschooler	305/1295 (23.6)	633/1694 (37.4)		
School-age	730/1224 (59.6)	1301/2322 (56.0)		
Season, n/N (%)			χ^2^ = 619.970	< 0.001
Winter	8/199 (4)	804/1966 (40.8)		
Spring	29/771 (3.7)	702/1902 (36.9)		
Summer	401/1301 (30.8)	625/1432 (43.6)		
Autumn	753/1939 (38.8)	115/683 (16.8)		
Clinical Severity, n/N (%)				
ICU Admission	6/234 (2.6)	20/321 (6.2)	χ^2^ = 4.074	0.044
Intervention, n/N (%)				
Bronchoscopy	29/40 (72.5)	205/243 (84.4)	χ^2^ = 3.376	0.066

Note: The Mann-Whitney U test (U) was used for age and the Chi-square test (χ²) for categorical variables. The surveillance periods were defined as December 2022–November 2023 and December 2023–November 2024. For Gender, Age group, and Season, percentages represent the proportion of MP-positive cases within each subgroup. For all other rows, percentages are based on the total number of patients in that period (*N* = 4210 and *N* = 5983)

### Analysis of clinical severity and interventions

We further compared clinical severity indicators between the two groups (Table 1). The MP-positive group showed a significantly lower proportion of ICU admissions compared to the MP-negative group (0.8% [26/3,437] vs. 7.8% [529/6,756]; *P* < 0.001). Conversely, the frequency of interventional fiberoptic bronchoscopy was higher in MP-positive patients (6.8% [234/3,437]) than in their MP-negative counterparts (0.7% [49/6,756]; *P* < 0.001). Notably, a temporal comparison revealed a shift in the contribution of MP to severe cases (Table 2). The proportion of ICU admissions associated with MP increased significantly from 2.6% (6/234) in the 2022–2023 period to 6.2% (20/321) in the 2023–2024 period (*P* = 0.044). Similarly, the proportion of bronchoscopies performed on MP-positive patients rose marginally from 72.5% to 84.4% (*P* = 0.066).

### Temporal alignment of co-detection frequency with overall respiratory viral burden

Longitudinal analysis revealed an apparent inverse temporal relationship between the monthly MP positivity rate and the viral co-detection rate among MP-positive patients; however, this correlation did not reach statistical significance (*R* = -0.19, *P* = 0.37, Fig. 1B-a). This dichotomy was most apparent when comparing distinct periods. During low MP circulation (December 2022–June 2023), MP positivity remained below 11%, while the viral co-detection rate was high, peaking at 75.0%. Conversely, as the MP epidemic surged (from July 2023), with MP positivity rates climbing to a peak of 48.2%, the co-detection rate decreased sharply, reaching a nadir of 11.3%.

In stark contrast, the rate of co-detection was strongly and significantly correlated with the overall monthly positivity rate of all other assayed viruses (*R* = 0.85, *P* = 1.4 × 10⁻⁷, Fig. 1B-b). This finding indicates that the probability of viral co-detection in an MP-positive patient is driven primarily by the community-level circulation of other respiratory viruses, rather than by the prevalence of MP itself. Months with higher overall viral prevalence were directly associated with a higher proportion of MP-virus co-detections.

### Prevalence and determinants of viral co-detection

**Fig. 4:**
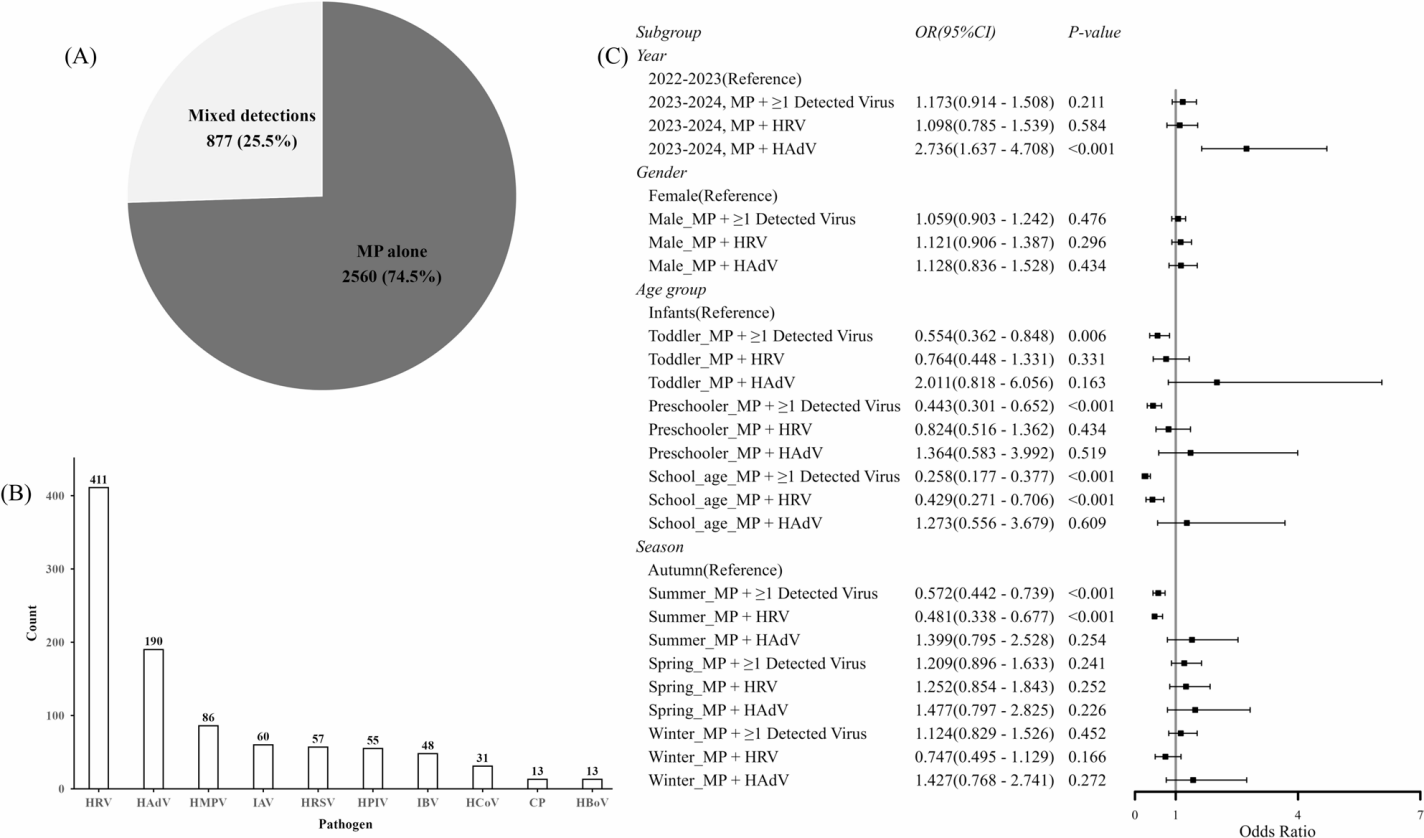
Characteristics and risk factor analysis of MP mixed detections. (**A**) Proportions of MP mono-detection (74.5%, *n* = 2560) versus co-detections (25.5%, *n* = 877). (**B**) Frequency of pathogens co-detected with MP in 877 patients. Note that the total number of detected pathogens (*n* = 964) exceeds the number of patients, as some individuals had multiple co-detections. (**C**) Forest plot from a multivariate logistic regression analysis showing odds ORs and 95% CIs for viral co-detection, stratified by subgroups (year, gender, age, and season)

Overall, viral co-detections were identified in 25.5% (877/3,437) of MP-positive patients. The most common co-detected pathogens were HRV and HAdV (Fig. 4A and B). Multivariable analysis confirmed that age was a primary determinant of co-detection, with risk decreasing progressively as age increased (Fig. 4C). Compared to the reference group of infants, school-age children had the lowest adjusted odds of viral co-detection (aOR 0.258, 95% CI: 0.177–0.377, *P* < 0.001). Furthermore, although the overall risk of co-detection remained unchanged between surveillance periods, we observed a specific and substantial increase in MP-HAdV co-detections during the 2023–2024 period (aOR 2.736, 95% CI: 1.637–4.708, *P* < 0.001).

## Discussion

This retrospective surveillance study provides compelling evidence for a fundamental paradigm shift in the epidemiology of MP during the post-COVID-19 era. Our findings demonstrate a transition from the canonical pattern of discrete, seasonal outbreaks skewed towards school-age children [11] to a sustained epidemic plateau, characterized by year-round, high-level transmission and a notable expansion of the susceptible age demographic. This transformation, defined by altered seasonality and evolving age-specific susceptibility [7, 12], challenges long-standing paradigms of MP circulation and mandates a recalibration of public health and clinical strategies for this resurgent pathogen.

Notably, we observed a marked transition from a canonical autumn-peaked epidemic wave in the first surveillance year to a prolonged plateau of high-level transmission throughout the second year. This pattern diverges significantly from established epidemiological trajectories for MP, not only within other regions of China [4, 12] but also from the broader global post-pandemic experience. In many other settings, the MP re-emergence was characterized by a synchronized, acute peak in late 2023 followed by a rapid decline [13]. While the initial 2023 resurgence in our cohort aligns with the prevailing “immunity debt” hypothesis [1, 2], the subsequent persistence of a high-transmission plateau in our coastal setting strongly suggests the involvement of additional, and potentially region-specific, drivers. We hypothesize that three factors may contribute to this “sustained plateau.” First, unique climatic conditions likely plays a role; the subtropical coastal climate involves prolonged periods of high relative humidity, which has been shown to enhance the environmental stability and transmission of MP [14]. Second, behavioral adaptations to high temperatures—specifically, the extensive use of air conditioning during the hot summer months—facilitate indoor crowding in enclosed spaces (e.g., shopping malls, schools), thereby mimicking the transmission dynamics typically seen in winter [15]. This pattern aligns with recent observations that MP infection is more prevalent in summer and autumn in southern China compared to the north [16]. Finally, as an economic hub with high population density, the region experiences frequent domestic migration and tourism, which can sustain local transmission dynamics and perpetuate the epidemic wave through constant re-introduction of pathogens [17].

Concomitant with this temporal shift, our study elucidates a critical evolution in the age-specific risk profile of MP [17]. Our analysis reaffirms the canonical age-dependent susceptibility to MP, wherein the risk escalates sharply after four years of age, peaking in school-aged children [12, 18]. Crucially, however, the risk curve for the 2023–2024 period exhibits a distinct leftward displacement, reflecting a significant expansion of susceptibility among toddler and preschool populations. This demographic expansion, a trend consistent with recent reports from other regions [7], challenges the conventional paradigm of MP as a pathogen primarily confined to school-aged children and necessitates the extension of diagnostic and preventive vigilance to younger age cohorts.

Our model further delineates a non-monotonic risk trajectory with age, which peaks at approximately 8.3 years before subsequently declining. This peak age of susceptibility aligns remarkably well with the median age of 8.5 years reported during concurrent MP resurgences in the United States and China [17, 19], thereby reinforcing the external validity of our findings. This observation has nuanced clinical implications. While school-aged children remain the primary risk group, the declining odds of MP infection in older pediatric cohorts suggest a shift in the etiological landscape of ARIs within this demographic. This observation raises the hypothesis that the relative contribution of other pathogens, such as influenza and adenoviruses, increases among older children presenting with ARIs. This finding underscores the necessity of a comprehensive, age-stratified diagnostic approach—a principle reinforced by extensive evidence of distinct pathogen profiles across pediatric age strata [18]. Such a strategy cautions against diagnostic anchoring on MP based on age alone, particularly for patients outside this peak-risk window.

Our analysis revealed a distinctive clinical profile for MP infection compared to MP-negative cases. Although MP-positive patients exhibited a lower rate of ICU admission (0.8%) than the MP-negative group (7.8%), they had a significantly higher requirement for interventional bronchoscopy (6.8% vs. 0.7%). This disparity may be attributed to the clinical characteristics of the disease and the demographics of the affected population. MP predominantly affects school-aged children and adolescents [11, 18]. Clinically, severe MP pneumonia frequently leads to airway obstruction via mucus plugs or plastic bronchitis [20] rather than immediate respiratory failure. Since older children generally possess larger airway calibers, they exhibit better physiological tolerance to such obstruction, resulting in fewer ICU admissions. In contrast, the requirement for intensive care is more commonly associated with younger children suffering from viral respiratory infections (e.g., RSV), who are more prone to rapid respiratory decompensation [18].

Viral co-detections were identified in 25.5% of MP-positive patients, predominantly comprising HRV and HAdV. Distinguishing true co-pathogenicity from asymptomatic colonization remains a challenge in molecular surveillance. Unlike typical bacterial colonizers (e.g., *S. pneumoniae*), MP exhibits significantly lower carriage rates in healthy children and is strongly associated with overt disease during epidemics [21], supporting its role as the primary etiological agent in this cohort. Conversely, viruses like HRV and HAdV can exhibit prolonged shedding after acute infection [22, 23]. Given the rigorous inclusion of strictly acute, hospitalized cases (symptom onset ≤ 14 days), we interpret these viral co-detections as likely representing clinically relevant co-infections; however, we retained the conservative term ‘co-detection’ throughout the text as this cannot be definitively confirmed without additional serological data. This etiological profile corroborates recent surveillance data from other major Chinese metropolitan areas [24–26]. Furthermore, in alignment with established pediatric epidemiology, the prevalence of viral co-detection exhibited a significant inverse correlation with age, reaching its zenith in infants [26, 27]. This trend presumably reflects the inherently higher baseline susceptibility to viral pathogens among younger cohorts rather than a specific synergistic interaction with MP. Of epidemiological note, however, was the marked statistical surge in MP-HAdV co-detections observed during the 2023–2024 epidemic period. Although the present study design precluded a direct assessment of clinical outcomes, prior investigations—particularly those from China—have consistently associated MP–adenovirus co-infection with exacerbated clinical severity [28–31].

Our ecological analysis suggests that these co-detections are primarily driven by concurrent high community transmission of respiratory viruses rather than MP prevalence itself. The strong correlation between ambient viral circulation and co-detection rates implies that MP occupies a distinct transmission niche and circulates largely uncoupled from the dynamics of seasonal viral epidemics [13]. Clinically, given the limited antiviral options for adenovirus [32], routine screening for co-pathogens may not be necessary for all MP-positive patients. Instead, we suggest that adenovirus testing be considered as part of multiplex PCR panels specifically for selected severe or refractory cases. In such instances, identifying co-detected pathogens is crucial to avoid attributing treatment failure solely to macrolide resistance [30, 31].

## Limitations

The findings of this study should be interpreted in light of several key limitations. First, our exclusive reliance on oropharyngeal swabs may not fully capture the etiological landscape of lower respiratory tract infections. Second, the single-center design may limit the generalizability of our findings, and the exclusion of both non-hospitalized and critically ill patients constrains our conclusions to a population with moderate-to-severe ARIs. Third, the study population was restricted to children aged ≤ 14 years due to the admission policies of our pediatric department; consequently, the epidemiological trends in older adolescents (15–18 years) remain uncharacterized in this cohort, which may influence the interpretation of the observed age shifts. Fourth, the absence of antimicrobial resistance data and detailed medication records limits the extent to which we can correlate pathogen detection with specific clinical courses, although associations with disease severity (ICU/bronchoscopy) were analyzed. Fifth, our reliance on RT-PCR detects nucleic acids rather than viable organisms, an inherent limitation that could lead to an overestimation of true infection prevalence. Finally, interpretations of the post-pandemic “resurgence” patterns should be tempered by potential fluctuations in case ascertainment and surveillance sensitivity. Prior evaluations of sentinel surveillance systems in Asia have documented temporary reductions in sensitivity and completeness during the acute COVID-19 period (2020–2021), followed by a recovery phase [33, 34]. Consequently, the observed sharp increase in MP positivity might partially reflect the restoration of healthcare-seeking behaviors and surveillance capacity following the public health emergency, rather than exclusively representing a biological resurgence of the pathogen. These limitations underscore the need for cautious interpretation but also define clear avenues for future research.

## Conclusion

This retrospective surveillance study describes a distinct shift in the post-pandemic epidemiology of MP within China’s subtropical coastal regions. The previously observed pattern of discrete seasonal outbreaks appears to have evolved into a sustained, year-round plateau of transmission, with an expanded susceptible demographic that includes younger, preschool-aged children. From a public health perspective, our findings highlight the potential benefit of moving from seasonal preparedness to continuous, year-round surveillance. Clinically, particularly when surveillance signals an upward trend in transmission, it is advisable to broaden the diagnostic scope to encompass all pediatric age groups, ensuring that toddlers and preschoolers are not overlooked. Moreover, the observed increase in MP–HAdV co-infections points to the importance of considering concurrent viral etiologies during diagnosis to ensure comprehensive patient assessment. In conclusion, while acknowledging its single-center scope, this study provides valuable regional data for understanding the evolving dynamics of MP. It underscores the need for further multicenter, integrated research to inform dynamic and evidence-based prevention and control strategies.

## Data Availability

The data that support the findings of this study are available from the corresponding author, upon reasonable request. The data are not publicly available due to ethical restrictions containing information that could compromise research participant privacy.

## Abbreviations

MP: Mycoplasma pneumoniae
ARIs: Acute Respiratory Infections
RT-PCR: Reverse transcription-polymerase chain reaction
Ct: Cycle Threshold
HPIV: human parainfluenza viruses
IVB: Influenza B virus
IVA: Influenza A virus
HAdV: human adenoviruses
CP: Chlamydia pneumoniae
HRV: Human rhinovirus
HBoV: Human bocavirus
HMPV: Human metapneumovirus
HCoV: Human coronaviruses
HRSV: Human respiratory syncytial virus
IQR: Interquartile range
RCS: Restricted Cubic Spline Regression
AIC: Akaike Information Criterion
BIC: Bayesian Information Criterion
ICU: Intensive care unit
Cis: Confidence intervals
COVID-19: Coronavirus disease 2019
